# Movements of Birds and Avian Influenza from Asia into Alaska

**DOI:** 10.3201/eid1304.061072

**Published:** 2007-04

**Authors:** Kevin Winker, Kevin G. McCracken, Daniel D. Gibson, Christin L. Pruett, Rose Meier, Falk Huettmann, Michael Wege, Irina V. Kulikova, Yuri N. Zhuravlev, Michael L. Perdue, Erica Spackman, David L. Suarez, David E. Swayne

**Affiliations:** * University of Alaska Museum, Fairbanks, Alaska, USA; † Institute of Arctic Biology, Fairbanks, Alaska, USA; ‡ Yukon Delta National Wildlife Refuge, Bethel, Alaska, USA; § Russian Academy of Sciences, Vladivostok, Russia; ¶ World Health Organization, Geneva, Switzerland; # United States Department of Agriculture, Athens, Georgia, USA

**Keywords:** avian influenza, migratory birds, influenza in birds, Asia, North America, research

## Abstract

Asian-origin avian influenza (AI) viruses are spread in part by migratory birds. In Alaska, diverse avian hosts from Asia and the Americas overlap in a region of intercontinental avifaunal mixing. This region is hypothesized to be a zone of Asia-to-America virus transfer because birds there can mingle in waters contaminated by wild-bird–origin AI viruses. Our 7 years of AI virus surveillance among waterfowl and shorebirds in this region (1998–2004; 8,254 samples) showed remarkably low infection rates (0.06%). Our findings suggest an Arctic effect on viral ecology, caused perhaps by low ecosystem productivity and low host densities relative to available water. Combined with a synthesis of avian diversity and abundance, intercontinental host movements, and genetic analyses, our results suggest that the risk and probably the frequency of intercontinental virus transfer in this region are relatively low.

## Abstract 2

Despite involvement of large numbers of birds, the delivery rate of Asian-origin viruses to North America through Alaska is apparently low.

In Alaska, diverse avian hosts from Asia and the Americas overlap in a region of intercontinental avifaunal mixing hypothesized to be an important zone of Asia-to-America virus transfer. Aquatic birds, especially waterfowl and shorebirds, provide a source of influenza viruses for transmission to mammals and poultry (*1**–**3*). Even without disease, when infected these avian hosts tend to shed high concentrations of virus in their feces (*4**–**6*). Cross-host infections in wild birds probably occur most frequently when other birds of the same or different species feed, drink, or bathe in waters contaminated by the feces of infected birds. On rare occasions, some of these avian influenza (AI) viruses, generally of low pathogenicity, have crossed species barriers from wild birds to poultry, in which mutations can produce highly pathogenic strains. From poultry, low and high pathogenicity viruses (or genomic segments of these viruses) can be introduced to humans, causing some fatal infections (*7*). This wild-bird reservoir can thus provide the genes for the next pandemic in humans or epizootic in domestic animals and presents an ongoing risk.

The rapid spread of highly pathogenic avian influenza A (H5N1) viruses from Asia across Eurasia (*8**,**9*) demonstrated how avian vectors can be involved in the distribution of avian and mammalian infections. Key activities for successful global influenza mitigation measures are surveillance, risk assessment, and epidemiologic modeling and prediction of AI virus infection in wild birds (*10**,**11*). Anthropogenic factors will also affect the evolution and distribution of avian influenza viruses. However, we focused on the natural virus transport system that migratory birds represent in an important high-latitude region with low levels of human presence.

We obtained our baseline data on viruses and vectors by screening wild birds for AI virus in western Alaska, starting in 1998. We focused on western Alaska because of the unparalleled overlap of Old World and New World bird migration systems in this region. To estimate the risk of Asian-origin AI viruses being delivered by migratory birds to North America through Alaska, we evaluated AI virus infection rates, bird movements, and the diversity and degree of intercontinental host overlap.

During the boreal summer, birds come to Alaska to breed from 6 continents: North and South America, Asia, Africa, Australia, and Antarctica. Alaska, thus, has direct, real-time connections with AI virus vectors from much of the world. It is a critical Old World–New World crossover zone and almost certainly a region of intercontinental virus transfer. Eurasian birds are common in Alaska during summer. Within 2 of the most important vector groups, waterfowl and shorebirds, at least 43 species regularly found in this region winter primarily in the eastern or southeastern parts of Asia; most are aquatic (Appendix Table). Additionally, many species are shared with Asia across the Bering Sea (Appendix Table). This extensive crossover of migratory Old World and New World birds offers excellent potential for virus exchange and transfer to the New World. Outbreaks of avian influenza that have killed persons in southeastern Asia (*7*) and occurrences of highly pathogenic AI (H5N1) infection in migratory birds (*8**,**9*) highlight the global importance of the Alaska migration system for intercontinental virus transport.

In Alaska, 5 major factors are involved in the natural intercontinental movement of avian-origin viruses: 1) Asian species coming to Alaska; 2) North American migrant birds (Asian-origin migrants that return to North America in autumn) breeding in Asia; 3) individual birds of species that winter on both sides of the Pacific moving between the continents; 4) species that have limited movements or are resident in the region; and 5) water (it has been inferred that live AI virus remains viable in fresh water at high latitudes through the cold northern winters) (*12*).

When we began our study, the role of migratory birds in the transport of highly pathogenic AI was uncertain, but wild birds have been found with these viruses, and with infection several species appear susceptible to severe disease and death (*9**,**13*). In previously reported cases of infection of wild birds with the highly pathogenic virus, transmission was thought to be from infected chickens, the species in which the shift to increased pathogenicity had originally occurred. Experimental and field studies (*9**,**14**,**15*) have identified highly pathogenic AI (H5N1) infection in ducks without clinical disease, which implicates healthy wild birds in transmission. Although in this regard the Asian AI (H5N1) subtype appears unique among highly pathogenic AI viruses, wild birds may be considered as potentially important vectors for strains of low and high pathogenicity.

## Methods

### Surveillance, Isolation, and Sequencing

We sampled ducks and shorebirds, 2 groups of aquatic birds important as subsistence foods in rural Alaska, widely associated with Alaska waters and common among those species that winter in southeastern Asia (Appendix Table). We sampled migratory birds that come to North America from southeastern Asian wintering grounds or Asian breeding grounds, as well as North American birds, which mingle with Old World birds on shared breeding and fall staging grounds. Intensive and extensive taxonomic sampling enabled us to obtain the best possible vectorwide prevalence estimates at our sampling sites. Our animal sampling was done according to protocols approved by the Institutional Animal Care and Use Committee.

 From May through October, 1998–2004, totals of 7,751 cloacal swabs and 503 fecal samples were collected from waterfowl (Anatidae) and shorebirds (Charadriidae and Scolopacidae), primarily at sites on the Yukon-Kuskokwim Delta, Alaska Peninsula, Seward Peninsula, the North Slope, and the Aleutian Islands. Most samples were obtained after the breeding season (Figure; Appendix Table). Each of these areas is internationally renowned for its avian diversity and abundance during the migration periods and the boreal summer.

Swabs and fresh fecal samples were placed in sterile medium (brain–heart infusion buffer with 10,000 U/mL penicillin G, 1 mg/mL gentamicin, 20 μg/mL amphotericin B) in the field and cooled before transport to the University of Alaska Museum laboratory. Transportation times were generally short (≈2–14 days), during which time samples were kept cool (mechanically refrigerated or buried above permafrost), frozen at –20°C, or kept on liquid nitrogen. Upon arrival at the laboratory, they were placed in a –70°C freezer. They were then shipped overnight to the Southeast Poultry Research Laboratory in Athens, Georgia, USA, in thick coolers with –70°C ice packs. Samples were not exposed to freeze-thaw cycles but were thawed for analysis.

Samples were processed for virus isolation (1998–2000) or screened by real-time reverse transcriptase–PCR (rRT-PCR) for influenza A virus (2001–2004), and all rRT-PCR–positive samples were subjected to virus isolation. Virus isolation was performed in embryonating chickens eggs as per standard procedures (*16*). For rRT-PCR, RNA was extracted from cloacal swab material with Trizol LS reagent (Invitrogen, Inc., Carlsbad, CA, USA) in accordance with manufacturer’s instructions. RNA was tested for avian influenza virus matrix (M) gene, which detects all type A influenza viruses (*17*). The recovery rate was no better when virus isolation was used (of the 5 isolates, 3 were from rRT-PCR and 2 were from direct virus isolation). Several thousand samples have been processed by rRT-PCR at the Southeast Poultry Research Laboratory with an internal positive control; the recovery rate between the methods is equivalent, which indicates that inhibition was not a major factor in our study.

### Mapping and Abundance

Delimitation of the Alaska portion of the overlap zone between Old World and New World migration systems in this region was done by using published and unpublished data on Alaska birds from the University of Alaska Museum. Abundance estimates were taken from published reports (*18**–**22*), books (*23*), and unpublished data (D.D.G., K.W., and University of Alaska Museum). Maps were created and species richness values were calculated by using ArcView 3.3 and ArcGIS 9.1 (Environmental Systems Research Institute, Inc., Redlands, CA, USA).

### Genetic Analyses

We used complementary population genetic approaches to assess approximate levels of individual intercontinental movement for 2 vector species of ducks: green-winged teal (*Anas crecca*) and mallards (*Anas platyrhynchos*). Gene flow between green-winged teal populations in eastern Asia (n = 14) and Alaska (n = 40) was determined by using amplified fragment-length polymorphisms (AFLPs). Whole genomic DNA was extracted from tissues by using the DNeasy Tissue Kit (QIAGEN, Valencia, CA, USA). AFLP data were generated by using the Applied Biosystems AFLP Plant Mapping Kit (Foster City, CA, USA) and protocol as described (*24*). Two fluorescently labeled primer pairs were run on an ABI PRISM 3100 Genetic Analyzer (Applied Biosystems). Electropherograms were scored manually and 218 loci were identified. STRUCTURE 2.1 (*25*) was used to indirectly estimate gene flow by using prior geographic information about each population and determining whether individuals were assigned genetically to their population of origin. Misassigned individuals are likely to have been immigrants or to have had recent immigrant ancestors (*25*). Because AFLPs provide dominant data, each locus is treated as a haploid allele; the no-admixture model with correlated allele frequencies was used. Multiple independent simulations were run at different lengths. Results are based on 100,000 burn-ins and 200,000 subsequent iterations. Pairwise *F_ST_* estimates (AFLP-SURV 1.0, [*26*]) gave comparable results (not shown). To estimate gene flow between mallards in Alaska (n = 39) and eastern Asia (n = 105), we used Migrate software (*27*) to estimate the neutral parameter theta (4*N_e_mµ*) and the migration rates between continents (4*N_e_m*) based on sequence data of 256 bp from intron 6 of ornithine decarboxylase (*28*). Multiple factors (e.g., mutation rates, effective vs. census population sizes, and percentage of immigrants successfully breeding) made it difficult to convert estimates based on population genetics to absolute numbers of immigrant individuals. Consequently, we chose a range of values commensurate with the moderate levels of gene flow found (Appendix Table).

### Estimating Asia-to-America Influenza Influxes

To estimate a baseline delivery rate of Asian-origin AI viruses to Alaska through these overlapping migration systems, we considered movement rates (*M*) of individuals from Asia (*i*) to Alaska (*j*) in conjunction with infection rates (*I*) and the incidence of specific influenza virus strains (*V_x_*) that we detected in this study. Measuring the risk associated with this threat thus becomes *M_ij_* × *I* = Asian-origin infected bird arrival; strain-specific incidence (*V_x_*) can be added to assess the narrower risk for subtypes, e.g., H5.

## Results

Within Alaska, the complexities of bird migration shape the taxonomic and geographic space where Asian-origin AI viruses are most likely to appear. Using Asian species as a guide, we coupled their distributions with those of American migrants (which are necessary to effectively transfer Asian AI virus to the greater New World) to define the extensive overlap of intercontinental avifaunas in northwestern North America (Figure) as the Beringian Crucible. Because of the mingling of intercontinental avifaunas, this area is most likely to harbor host switching and genetic reassortment among AI viruses from Asia and the Americas.

**Figure:**
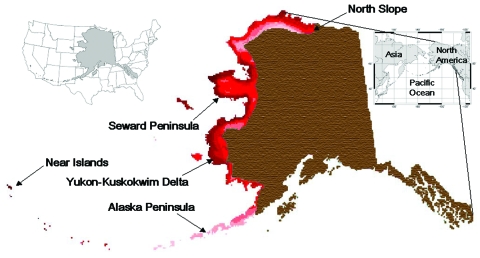
Composite geographic information system map illustrating the overlap of New World and Old World migration systems among 64 species of waterfowl (family Anatidae) and shorebirds (families Charadriidae and Scolopacidae) in northern and western Alaska (darkness of shade indicates species richness). This overlap between Asiatic and American birds in these families occurs in a zone whose extent is equivalent to a geographic band running from Lake Superior to North Dakota then to Texas and California in the lower 48 US states (left inset).

Our surveillance of wild-bird AI virus focused on the eastern, or North American, part of the Beringian Crucible (Figure). We found low rates of infection among the 8,254 samples obtained from the most important host groups, waterfowl (Anatidae) and shorebirds (Charadriidae and Scolopacidae; Appendix Table). From these samples we obtained only 5 isolates, which represent an infection rate of just 0.061%. These isolates included hemagglutinin subtypes H3, H4, and H6 (*29*). The 5 isolates were found in 3 (0.2%) of 1,477 green-winged teal (*Anas crecca*), 1 (0.76%) of 131 mallards (*Anas platyrhynchos*), and 1 (0.03%) of 3,703 northern pintails (*Anas acuta*). We found neither evidence of a clearly Eurasian origin for any of the virus genes sequenced from these Alaska isolates (*29*) nor H5 subtypes. Our data do show a remarkably close genetic association between avian influenza (H6) virus in Alaska ducks and a poultry outbreak in California in nucleoprotein and nonstructural protein A genes (*29*). This finding reflects real-time connections of migratory ducks between Alaska and California, and this vector connection extends into the Russian Far East (*30*). These findings affirm the intracontinental importance and risk posed by this region.

The numbers of individuals of the most important host groups (waterfowl and shorebirds) that come to Alaska from Asia is an important and heretofore unknown variable that affects the level of risk posed by these birds. Asian species are easiest to enumerate, because species-level identity indicates origin. However, many key vector species occur on both sides of the North Pacific and move regularly between Asia and North America (Appendix Table) and thus represent another important group of species for risk assessment. Within-species intercontinental movements of taxa that are distributed across both Asia and North America are challenging to quantify. Most species-level information is inadequate, and methods such as bird banding have not provided numeric estimates of these movements. We have summarized available data and used population genetics in 2 key vector species to estimate degrees of intercontinental avifaunal interchange in this region (Appendix Table; an expanded version is available from the authors). Our population genetic work used 2 complementary methods and focused on 2 duck species carrying AI viruses in this region. For green-winged teal, assignment tests using AFLP markers showed that ≈2 (5%) of 40 individuals from Alaska appeared to be recent immigrants from Asia. In mallards, migration-rate values (4*N_e_m*, the number of immigrants in relation to effective population size) for individuals coming from Asia to Alaska were 1,064−1,727 (95% confidence interval) effective immigrants per generation. In each of these host species, intercontinental gene flow thus appears to be moderate (neither very low nor high), which indicates that thousands of individuals of these species may be coming to Alaska from Asia each year (Appendix Table). These results corroborate the limited observational evidence from which we understood these movements to be well above zero but not high.

We estimate that 1.5–2.3 million birds from the families Anatidae, Charadriidae, and Scolopacidae come to Alaska from Asia each year (Appendix Table). Multiplying this vector flow by the 0.061% AI infection rate that we measured among these families in Alaska suggests that 901–1,389 Asian-origin viruses may come from this source. However, our measure of infection rates is based on ducks in autumn, a taxonomic group and time known for increased infection rates (*31**,**32*). Although a few of our autumn duck samples are probably from birds coming from Asian breeding grounds, we have no isolates from Scolopacidae, perhaps due to fewer samples. Scolopacidae is the numerically dominant host group and is more likely to bring Asian-origin viruses in spring (Appendix Table, [*32*]). Thus, our estimates of virus coming to Alaska from Asia can be considered to be high. Asian-origin AI virus arrival would most likely occur in the Beringian Crucible (Figure), which in western Alaska is 256,400 km^2^, about the size of the United Kingdom or the US states of Wyoming or Oregon. Insofar as we have not detected H5 or H7 subtypes among our 8,254 samples, their incidence has been too low to effectively measure. Given the statistical power of our sample, their delivery rate from Asia through this system appears to be very low.

## Discussion

Our surveillance did not show a “hotspot” of AI virus infection among avian hosts. Much higher infection rates are known from other multiyear surveillance studies at lower latitudes, e.g., Delaware Bay (≈4.7%, [*32*]), southern Minnesota (10.8%, [*5*]), and Alberta (22.2%, [*32*]) and British Columbia in Canada (55%, although only a single-year study, [*33*]). The infection rates we found are substantially lower than those found for interior Alaska (9%, [*12*]). Arctic conditions in Alaska prevail well south of the Arctic Circle in the treeless regions of western Alaska, and the US Arctic includes the Alaska Peninsula and Aleutian Archipelago (*34*), a tundra ecosystem where our sampling was concentrated (Figure). Aerial surveys of waterfowl across Alaska show more ponds and fewer ducks per unit area on tundra; the number of ducks per pond on tundra habitat (2.1) is less than half the number found in the boreal-forest dominated interior (5.5, [*18*]). This simple ecologic factor (perhaps due to the lower productivity of these tundra ecosystems), resulting in the dilution of virus in waters with fewer available hosts, may in part explain our results. This is the first geographically and taxonomically extensive Arctic AI surveillance in North America, and it suggests that some Arctic effect lowers infection rates, thus lowering the risk of intercontinental viral transfer in these high-latitude regions. Our infection rates are low, comparable to those occurring at much lower latitudes (e.g., *9**,**35*), whereas mid-latitude rates can be 2–3 orders of magnitude higher (*33*).

Human population densities in Alaska are relatively low, especially in the Beringian Crucible, and Alaska lacks a large agricultural sector. However, mammalian carnivores abound and could be susceptible hosts (*36*). Direct human infection from wild birds is possible, but transmission from birds to humans is difficult (*37**,**38*). Nevertheless, exposure in this region may be considerable; hunters kill ≈99,000 waterbirds for food each year on the Yukon-Kuskokwim Delta alone (*39*).

Although the existence of North American and Eurasian viral lineages is well established in the literature, evidence from other regions of North America has shown that geographic structure has been insufficient to prevent sporadic intercontinental exchange of some hemagglutinin subtypes (*29**,**40*). Our results can be considered to confirm the comparative rarity of such events in this important region of Alaska. Despite high diversity of host species and high numbers of individual birds in Alaska making intercontinental movements, the low AI infection rates and the genetic attributes of virus isolates (*29*) suggest that at most only small numbers of Asian-origin AI viruses or genes likely arrive in Alaska annually. Although AI viruses from Alaska have a clear link with other viruses in the lower 48 US states (*29*), the predominance of Arctic ecologic conditions and the lack of agriculture in the Alaska region most affected suggest a low risk for intercontinental viral transfer in this region.

## Supplementary Material

Appendix Table Species of waterfowl (Anatidae) and shorebirds (Charadriidae, Recurvirostridae, and Scolopacidae) in Alaska with an Old World connection or from which cloacal swabs or fecal samples were obtained, Alaska, 1998-2004*
