# Supplementary material for: Movements of Birds and Avian Influenza from Asia into Alaska
Source: Emerg Infect Dis. 2007 Apr;13(4):547–52. doi: 10.3201/eid1304.061072 (PMC2725966; doi:10.3201/eid1304.061072)
Supplement: Appendix Table — Species of waterfowl (Anatidae) and shorebirds (Charadriidae, Recurvirostridae, and Scolopacidae) in Alaska with an Old World connection or from which cloacal swabs or fecal samples were obtained, Alaska, 1998-2004* [file 06-1072_appT-s1.pdf]

Publisher: CDC; Journal: Emerging Infectious Diseases
Article Type: Research; Volume: 13; Issue: 4; Year: 2007; Article ID: 06-1072
DOI: 10.3201/eid1304.061072; TOC Head: Research

Appendix Table. Species of waterfowl (Anatidae) and shorebirds (Charadriidae, Recurvirostridae, and Scolopacidae) in Alaska with an Old World connection or from which cloacal swabs or fecal samples were obtained, Alaska, 1998–2004*

Common name	Taxonomic name	No. swabs	OW min	OW max	Source(s)
**Bean goose**	***Anser fabalis***	0	100	100	Authors
Greater white-fronted goose	*Anser albifrons*	0	3,000†	30,000	(1,2)
**Lesser white-fronted goose**	***Anser erythropus***	0	10	10	Authors
Emperor goose	*Chen canagica*	17	8,625	11,500	(3)
Snow goose	*Chen caerulescens*	0	80,000	80,000	(3)
Brant	*Branta bernicla*	6	510‡	2,550‡	(4)
Cackling goose	*Branta hutchinsii*	443			
Tundra swan	*Cygnus columbianus*	0	1,420†	14,200	(3)
**Whooper swan**	***Cygnus cygnus***	0	500	500	Authors
Gadwall	*Anas strepera*	1	50†	500	Authors
**Falcated duck**	***Anas falcata***	2	50	50	Authors
**Eurasian wigeon**	***Anas penelope***	8	800	800	Authors
American wigeon	*Anas americana*	7			
Mallard	*Anas platyrhynchos*	131	7,000§	35,000§	(5)
**Spot-billed duck**	***Anas poecilorhyncha***	0	10	10	Authors
Northern shoveler	*Anas clypeata*	17	6,660†	66,600	(5)
Northern pintail	*Anas acuta*	3,703	45,250†	135,750	(5)
**Garganey**	***Anas querquedula***	1	50	50	Authors
**Baikal teal**	***Anas formosa***	1	50	50	Authors
Green-winged teal	*Anas crecca*	1,477	7,130§	35,650§	(5)
Anas sp.	*Anas acuta, crecca,* or *platyrhynchos*	610			
Canvasback	*Aythya valisineria*	1			
Redhead	*Aytha americana*	2			
**Common oochard**	***Aytha ferina***	0	50	50	Authors
Ring-necked duck	*Aytha collaris*	5			
**Tufted duck**	***Aytha fuligula***	2	450	500	Authors
Greater scaup	*Ayatha marila*	53	9,000†	90,000	(5)
Lesser scaup	*Aytha affinis*	29			
Steller's eider	*Polysticta stelleri*	615	410†	4,100	(3,5)
Spectacled eider	*Somateria fischeri*	0	12,000	12,000	(3)
King eider	*Somateria spectabilis*	1	500†	3,500	(3)
Common eider	*Somateria mollissima*	18	255‡	1,275‡	(3)
Harlequin duck	*Histrionicus histrionicus*	9	1,700‡	8,500‡	(3), Authors
Surf scoter	*Melanitta perspicillata*	1			
White-winged scoter	*Melanitta fusca*	0	875‡	4,375‡	(5), Authors

Black scoter	*Melanitta nigra*	0	1,400‡	7,000‡	(5), Authors
Long-tailed duck	*Clangula hyemalis*	2	660‡	3,300‡	(5), Authors
Bufflehead	*Bucephala albeola*	3			
Common goldeneye	*Bucephala clangula*	1	1,500†	15,000	(5), Authors
**Smew**	***Mergellus albellus***	0	50	50	Authors
Common merganser	*Mergus merganser*	3	100†	500	Authors
Red-breasted merganser	*Mergus serrator*	10	360‡	1,800‡	(5)
Black-bellied plover	*Pluvialis squatarola*	11	1,000	5,000	(6), Authors
**European golden plover**	***Pluvialis apricaria***	0	10	10	Authors
American golden plover	*Pluvialis dominica*	26			
Pacific golden plover	*Pluvialis fulva*	39	500†	4,000	(6), Authors
**Lesser sand plover**	***Charadrius mongolus***	4	500	500	Authors
Snowy plover	*Charadrius alexandrinus*	0	10	0	Authors
**Common ringed plover**	***Charadrius hiaticula***	1	50	50	Authors
Semipalmated plover	*Charadrius semipalmatus*	19			
**Little ringed plover**	***Charadrius dubius***	0	10	10	Authors
**Eurasian dotterel**	***Charadrius morinellus***	0	100	100	Authors
**Black-winged stilt**	***Himantopus himantopus***	0	10	10	Authors
**Common greenshank**	***Tringa nebularia***	6	250	250	Authors
Greater yellowlegs	*Tringa melanoleuca*	16			
Lesser yellowlegs	*Tringa flavipes*	4			
**Marsh sandpiper**	***Tringa stagnatilis***	0	10	10	Authors
**Spotted redshank**	***Tringa erythropus***	0	50	50	Authors
**Wood sandpiper**	***Tringa glareola***	35	500	500	Authors
**Green sandpiper**	***Tringa ochropus***	0	10	10	Authors
Solitary sandpiper	*Tringa solitaria*	1			
Wandering tattler	*Heteroscelus incanus*	24	200†	2,000	(7)
**Gray-tailed tattler**	***Heteroscelus brevipes***	20	500	500	Authors
**Common sandpiper**	***Actitis hypoleucos***	5	100	100	Authors
Spotted sandpiper	*Actitis macularius*	3			
**Terek sandpiper**	***Xenus cinereus***	2	50	50	Authors
**Little curlew**	***Numenius minutus***	0	10	10	Authors
Whimbrel	*Numenius phaeopus*	21	1,050	1,050	(6), Authors
Bristle-thighed curlew	*Numenius tahitiensis*	1			
**Far Eastern curlew**	***Numenius madagascariensis***	0	25	25	Authors
**Black-tailed godwit**	***Limosa limosa***	2	25	25	Authors

Hudsonian godwit	*Limosa haemastica*	6			
**Bar-tailed godwit**	***Limosa lapponica***	49	120,000	120,000	(6)
Marbled godwit	*Limosa fedoa*	14			
Ruddy turnstone	*Arenaria interpres*	50	2,500	3,750	(6)
Black turnstone	*Arenaria melanocaphala*	19			
Surfbird	*Aphriza virgata*	10			
**Great knot**	***Calidris tenuirostris***	0	25	25	Authors
**Red knot**	***Calidris canutus***	13	47,500	47,500	(6)
Sanderling	*Calidris alba*	3	30†	450	(6), Authors
Semipalmated sandpiper	*Calidris pusilla*	37			
Western sandpiper	*Calidris mauri*	79	36,750†	351,750	(6)
**Red-necked stint**	***Calidris ruficollis***	12	1,000	1,000	Authors
**Little stint**	***Calidris minuta***	1	25	25	Authors
**Temminck's stint**	***Calidris temminckii***	2	100	100	Authors
**Long-toed stint**	***Calidris subminuta***	9	100	100	Authors
Least sandpiper	*Calidris minutilla*	10			
Baird's sandpiper	*Calidris bairdii*	11	750	750	(6), Authors
Pectoral sandpiper	*Calidris melanotos*	34	20,000	20,000	(6), Authors
**Sharp-tailed sandpiper**	***Calidris acuminata***	11	7,500	10,000	(6)
Rock sandpiper	*Calidris ptilocnemis*	237	7,500†	30,000	(6)
Dunlin	*Calidris alpina*	37	648,000	648,000	(6)
Stilt sandpiper	*Calidris himantopus*	13			
**Curlew sandpiper**	***Calidris ferruginea***	0	25	25	Authors
**Spoon-billed sandpiper**	***Eurynorhynchus pygmeus***	0	10	10	Authors
**Broad-billed sandpiper**	***Limicola falcinellus***	0	25	25	Authors
Buff-breasted sandpiper	*Tryngites subruficollis*	20			
**Ruff**	***Philomachus pugnax***	0	250	250	Authors
Short-billed dowitcher	*Limnodromus griseus*	2			
Long-billed dowitcher	*Limnodromus scolopaceus*	26	17,500†	52,500	(3)
**Jack snipe**	***Lymnocryptes minimus***	0	10	10	Authors
Wilson's snipe	*Gallinago delicata*	13			
**Common snipe**	***G. gallinago***	11	1,000	1,000	Authors
**Pin-tailed snipe**	***G. stenura***	1	25	25	Authors
Red-necked phalarope	*Phalaropus lobatus*	26	5,000†	50,000	(3,6)
Red phalarope	*Phalaropus fulicarius*	26	375,000	375,000	(3,6)
Feces of ducks and shorebirds		53			
Total swabs and fecal		8,254			

samples		
**Total minimums and maximums**	**1,486,120**	**2,291,825**
Anatidae	190,525	565,270
Charadriidae	2,180	9,670
Scolopacidae	1,293,415	1,716,885

*For those species with an Old World connection, we provide estimates of the numbers of individuals that come to Alaska annually from the Old World. OW min, minimum from Old World; OW max, maximum from Old World. Species in **boldface** (43 total) are Asian; others (37 additional species, those for which intercontinental population estimates are given) are shared between the New World and Old World.
†Lower limits estimated.
‡Extrapolated assuming that population genetic estimates from 2 anatid species are representative.
§Estimated from population genetics using 1%–5% of Alaska population moving between continents as sufficient to cause the moderate levels of gene flow observed.

Appendix Table References

1. Eldridge W, Hodges J, Bollinger K. Report to the Pacific Flyway Committee on the 1985–2005 Coastal Zone Yukon-Kuskokwim Delta goose survey of geese, swans, and sandhill cranes. Anchorage (AK); US Fish and Wildlife Service; 2005.

2. Nieman D, Warner WK, Smith J, Solberg J, Roetker F, Lobpries D, et al. Fall inventory of mid-continent white-fronted geese, 2004 [report]. Saskatoon (Saskatchewan): Canadian Wildlife Service; 2004.

3. Poole A, Gill F, eds. The birds of North America online. [cited 2006 Apr 12]. Available from http://bna.birds.cornell.edu/BNA

4. Conant B, King RJ. Winter waterfowl survey: Mexico west coast and Baja California. Juneau (AK): US Fish and Wildlife Service; 2006.

5. Conant B, Groves DJ. Alaska-Yukon waterfowl breeding population survey [report]. Juneau (AK): US Fish and Wildlife Service; 2005.

6. Alaska Shorebird Working Group. A conservation plan for Alaska shorebirds. Anchorage (AK): US Fish & Wildlife Service and US Geological Survey; 2006.

7. Shinya K, Ebina M, Yamada S, Ono M, Kasai N, Kawaoka Y. Influenza virus receptors in the human airway. Nature. 2006;440:435–6.
